# Benign nerve tumours in the upper limb: a registry-based study of symptoms and surgical outcome

**DOI:** 10.1038/s41598-023-38184-9

**Published:** 2023-07-17

**Authors:** Emanuel Istefan, Malin Zimmerman, Lars B. Dahlin, Erika Nyman

**Affiliations:** 1 Department of Biomedical and Clinical Sciences, Linköping University, Linköping, Sweden; 2 Department of Orthopaedics, Helsingborg Hospital, Helsingborg, Sweden; 3 Department of Translational Medicine – Hand Surgery, Lund University, Malmö, Sweden; 4 Department of Hand Surgery, Skåne University Hospital, Malmö, Sweden; 5 Department of Hand Surgery, Plastic Surgery and Burns, Department of Biomedical and Clinical Sciences, Linköping University Hospital, 581 83 Linköping, Sweden

**Keywords:** Outcomes research, Neurological manifestations

## Abstract

Surgery for benign nerve tumours is performed for pathoanatomical diagnosis and symptomatic relief, but might cause residual problems. We aimed to assess patient-reported symptoms and disability before and after surgery at a national level. In total, 206 cases surgically treated for a benign peripheral nerve tumour 2010–2019 registered in the Swedish Quality Registry for Hand Surgery (HAKIR; response rates 22–34%) were analysed. Surgery reduced overall disability in the affected limb (QuickDASH 18/100 [IQR 5–36] preoperatively and 5/100 [IQR 0–22] 12 months postoperatively), improved ability to perform daily activities (HQ-8; 11/100 [IQR 0–50] preoperatively and 0/100 [IQR 0–20] 12 months postoperatively) and decreased three evaluated pain modalities: pain at rest (HQ-8; 20/100 [IQR 0–40] preoperatively and 0/100 [IQR 0–10] 12 months postoperatively), pain on motion without load (HQ-8; 20/100 [IQR 0–40] preoperatively and 0/100 [IQR 0–10] 12 months postoperatively), and pain on load (HQ-8; 24/100 [IQR 1–69] preoperatively and 1/100 [IQR 0–30] 12 months postoperatively). Cold sensitivity was a minor problem both before and after surgery (HQ-8; 0/100 [IQR 0–30] preoperatively and 1/100 [IQR 0–40] 12 months postoperatively). We conclude that surgery for benign peripheral nerve tumours provides good symptomatic relief with low risk for residual problems.

## Introduction

Benign nerve tumours are a heterogeneous group usually originating from the nerve sheath. Most common are Schwannomas, followed by neurofibromas, perineuriomas and lipofibromatous hamartomas^1–5^. The mean incidence of Schwannomas in the upper limb requiring surgical treatment has been approximated to 0.62 per 100 000 inhabitants and year^2^. Schwannomas are slow-growing tumours and tend to cause symptoms, such as pain, paraesthesia, and motor dysfunction as well as imposing disability, when they get larger^2,6–8^. This can be explained by the mass expansion of the tumour and limited resilience in the surrounding tissue, resulting in extrinsic compression of healthy nerve fibres^9,10^.

Surgical treatment with excision is recommended for Schwannomas and neurofibromas causing pain or other symptoms^2,11^. Microsurgical technique is recommended to minimise the risk of damaging healthy nerve fibres^4,8,12–14^. Damage of the nerve fibres directly affected by the Schwannoma cannot always be avoided as the configuration of nerve tumours may incorporate nerve fibres or off-shooting nerve branches. Furthermore, surgery may also harm surrounding healthy nerve fibres in the fascicles bordering the tumour by the longitudinal incision to the epineurium, or intact fascicles not involved in the tumour due to compression or damage during the operation^15^.

Following surgical treatment of nerve tumours, temporary postoperative symptoms, such as pain, paraesthesia, and sensory dysfunction, may be present, but permanent symptoms are reported as unusual^12,15–17^. Cold sensitivity, with discomfort on exposure to cold, is common after hand injuries, particularly nerve injuries, and in nerve compression disorders^18–22^. However, less is known about cold sensitivity following excision of benign peripheral nerve tumours.

Due to the rarity of benign peripheral nerve tumours, the number of large descriptive and comparative studies are limited^7^. The literature is also sparse regarding surgical outcome in larger populations and greater follow-up studies are needed to evaluate patients surgically treated for benign peripheral nerve tumours in the upper limb. The aim of this study was to assess patient-reported symptoms and disability before and after surgical intervention at a national level.

## Methods

### Overview

This is a prospective register-based cohort study with an observational interpretation of patient-reported outcome measures (PROMs) amongst patients surgically treated for benign nerve tumours in the upper limb utilising prospectively collected data from the Swedish Quality Registry for Hand Surgery (HAKIR, www.hakir.se). Patients, identified with the ICD-10 (International Statistical Classification of Diseases and Related Health Problems, revision 10) code D36.1, representing benign neoplasms of peripheral nerves and the autonomic nervous system, were included from HAKIR^23^ between January 1st 2010 and December 31st 2019.

### Participants

All patients above 16 years old having surgery at any of the seven specialized departments of hand surgery in university hospitals, together with three private healthcare providers (at that time) in Sweden, are included in the registry. No exclusion criteria were applied beyond those implemented at the enrolment in HAKIR, namely: cognitive problems, reoperation within 1 year, lack of a Swedish social security number, and declined participation in the registry^24^. The data variables gathered from HAKIR were sex, age, date of surgery, if a re-operation was performed with complementary reasons for the reoperation, primary and secondary code according to ICD-10, primary and secondary surgical code, number of operations, dominant hand, and which hand was surgically treated as left, right or both. Before surgery, and at three and 12 months after surgery the questionnaires Quick Disabilities of the Arm, Shoulder and Hand (QuickDASH) and the eight-item HAKIR questionnaire (HQ-8) were filled in by the patients^24^. The nerve and the level of surgical engagement, based on available Swedish version of the NOMESCO Classification of Surgical Procedures (NCSP-S) that classifies health-care measures or interventions, as defined by the National Board of Health and Welfare (Socialstyrelsen.se), were recorded at data entry at surgery into the register and then collected directly from the HAKIR register. Concerning level of surgery, this could not be determined for the applicable NCSP-S code (ACB19); thus, rendering surgery other than the defined levels hand and wrist, forearm, and elbow, as well as upper arm and shoulder as uncertain level. In addition, there are no specific NCSP-S codes for surgeries targeting digital and common digital nerves, as well as other minor nerves in the hand and arm. Therefore, other nerves than the major nerve trunks, i.e., median, radial, and ulnar nerves, were defined as “other nerves”.

### Patient-reported outcome measures (PROMs)

The QuickDASH score assesses symptoms and disability in the upper limb, ranging from 0, representing no impairment, to 100, indicating maximal impairment. Besides the calculated total QuickDASH score, the seventh and eleventh items in the QuickDASH questionnaire were utilised separately to extrapolate impairment of social life and sleep-related problems.

HQ-8 is an eight-item questionnaire developed by and exclusive to HAKIR^24^. HQ-8 concerns only the patient’s affected hand and addresses different symptoms with seven questions regarding perceived pain at rest, pain on motion without load, pain on load, stiffness, weakness, numbness, and cold sensitivity. The last item accounts for the ability to perform daily activities with the affected hand. Additionally, HQ-8 provides two supplementary and optional items evaluating overall patient satisfaction of the operation and the received care at the clinic during treatment. All HQ-8 items are also scaled from 0, representing no impairment or maximal satisfaction, to 100, representing maximal difficulties or dissatisfaction. Data completeness in HQ-8 is good, with an expected floor effect, lack of ceiling effect and the ability to detect changes in items over time^24^.

### Statistical methods

Data are presented as the number of observations and the percentage of all valid observations. Variables that were normally distributed are presented as mean with standard deviation (SD), otherwise the median is presented with the interquartile range [IQR] as the 25th to the 75th percentile. No outcome measures were normally distributed. Differences between time points were analysed with the Kruskal–Wallis test and the Mann–Whitney U test in associated post hoc analyses. Multiple correlation analyses between all items in the HQ-8 questionnaire together with items 7 and 11 in the QuickDASH questionnaire were performed before and at 12 months after surgery utilising Spearman’s correlation test with False Discovery Rate (FDR) correction according to the Benjamini–Hochberg procedure. Correlations were considered weak if the correlation coefficient was less than 0.5, moderate if the correlation coefficient ranged between 0.5 and 0.69, and strong if the coefficient was equal or greater than 0.7. The HQ-8 questionnaire and its items are designed for item-separate analyses, and the items are not compiled into a general score^24^.With regards to QuickDASH, similar sub-analyses have previously been performed^22^.

Differences in cold sensitivity (item 7 in the HQ-8 questionnaire) were analysed preoperatively compared to 12 months postoperatively and with regards to the seasons of the year defined as warmer months (April through September) or colder months (October through March) during a year in Sweden.

### Ethics declarations

Ethical approval was obtained from the Swedish Ethical Review Authority (Dnr 2020-01484). Informed consent was obtained at the time of inclusion to HAKIR. The study was planned and conducted according to the Declaration of Helsinki (7th revision 2013).

### Informed consent

Written informed consent was obtained from all subjects before this study at the time of inclusion in HAKIR.

## Results

### Patient characteristics

Patient characteristics are presented in Table 1. The population includes 206 cases from 197 patients surgically treated for a benign peripheral nerve tumour in the upper limb. The overall sex distribution was rather equal with a mean age of 50 (SD 16) years. The level of surgery in the upper limb was derivable from the given surgical code (NCSP-S) in 55/206 (27%) cases, while in 151/206 (73%) the level of engagement was uncertain. Of the 55 cases with defined level, the tumours were most frequently located in the hand or wrist (38/55; 69%). Data on affected nerve was derivable from the given surgical codes (NCSP-S) in 73/206 (35%) cases, while in 133/206 (64%) cases other nerves were affected. Of those 73 cases, the tumour was mostly located in the ulnar (32/73, 44%) or the median (29/73, 40%) nerves.

**Table 1:** Demographic data on patients surgically treated for peripheral nerve tumours in the upper limb.

	Total n (%) (n = 206)
Sex (F:M)	95:102 (48:52)
Mean age	50 (SD 16)
Level of surgery	
Hand & wrist	38 (18)
Forearm & elbow	4 (2)
Upper arm & shoulder	13 (6)
Uncertain level	151 (73)
Engaged nerve	
Median nerve	29 (14)
Ulnar nerve	32 (16)
Radial nerve	12 (6)
Other nerves	133 (64)
Surgery on dominant hand (n = 114)	63 (55)
Revisions^a^	20 (10)

Age is presented in years. Uncertain level of surgery is defined as surgery other than and not defined as the other three levels. Engagement of other nerves are defined as digital and common digital nerves, as well as other minor nerves in the hand and arm. *Abbreviations* F, Female; M, Male; SD, Standard deviation.

^a^indicates that two of these patients were included in the outcome analyses presented in Table 2. The two reoperations were performed due to lack of surgical competence at initial surgery; thus, not strictly defined as a reoperation.

A reoperation was performed in 20/206 (10%) cases due to recurrence of a neoplasm (4/20), neuroma formation (2/20), isolated neuralgia (1/20), restricted primary procedure due to limited surgical experience (2/20), and other unknown reasons (11/20). Only two of these patients were included in the outcome analyses since these two reoperations were performed due to lack of surgical competence at the initial surgical procedure (i.e., primary exploration of tumour and immediate closure of skin; thus, not a reoperation in its strict definition).

### QuickDASH total score and HQ-8 before and after surgery

**Figure 1:**
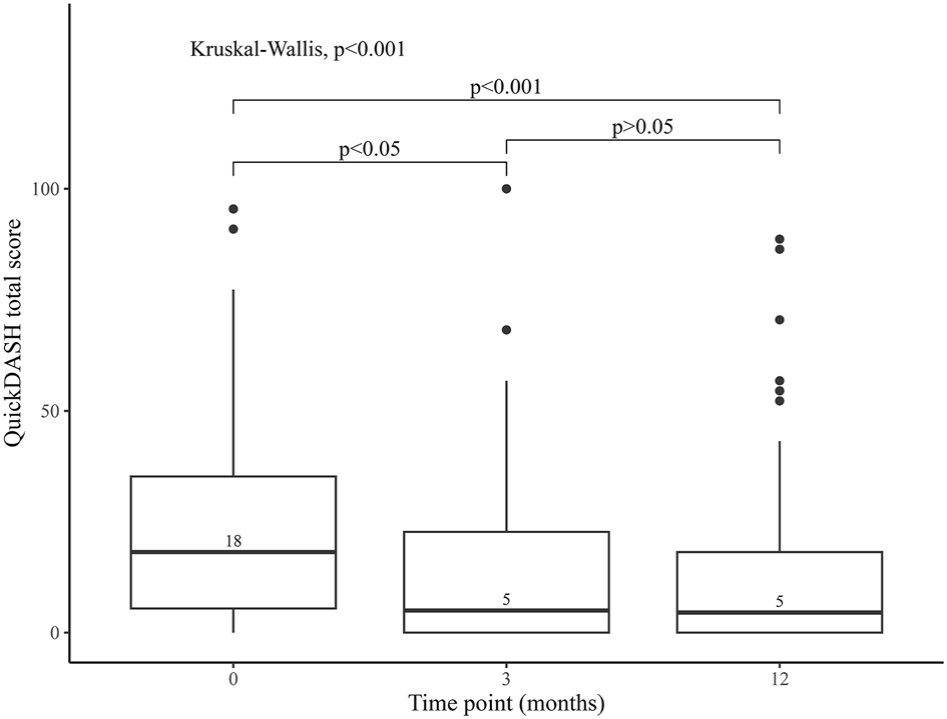
Boxplot presenting QuickDASH total score between different time points in months relative to surgery with overall *p*-value corresponding to the Kruskal–Wallis test. *p*-values from the post hoc Mann–Whitney U tests are associated with brackets indicating compared groups. *Abbreviations* QuickDASH, Quick Disabilities of the Arm, Shoulder and Hand.

**Figure 2:**
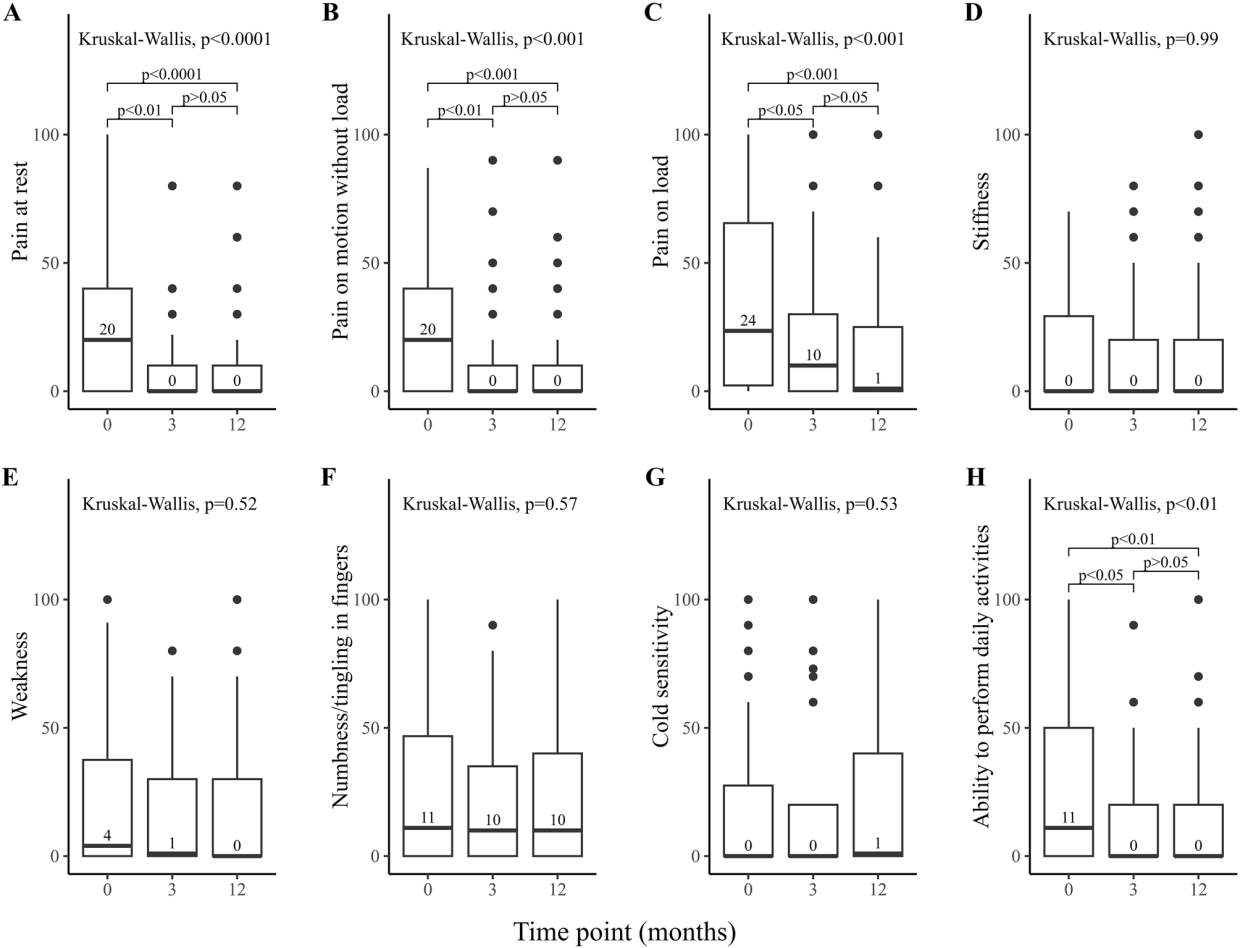
HQ-8 questionnaire items presented as boxplots at the different time points with *p*-values from matching Kruskal–Wallis test. *p*-values from post hoc Mann–Whitney U tests are associated with brackets indicating compared groups. (**A**) Pain at rest; (**B**) Pain on motion without load; (**C**) Pain on load; (**D**) Stiffness; (**E**): Weakness; (**F**): Numbness/tingling in fingers; (**G**): Cold sensitivity (discomfort on exposure to cold); (**H**): Ability to perform daily activities.

**Figure 3:**
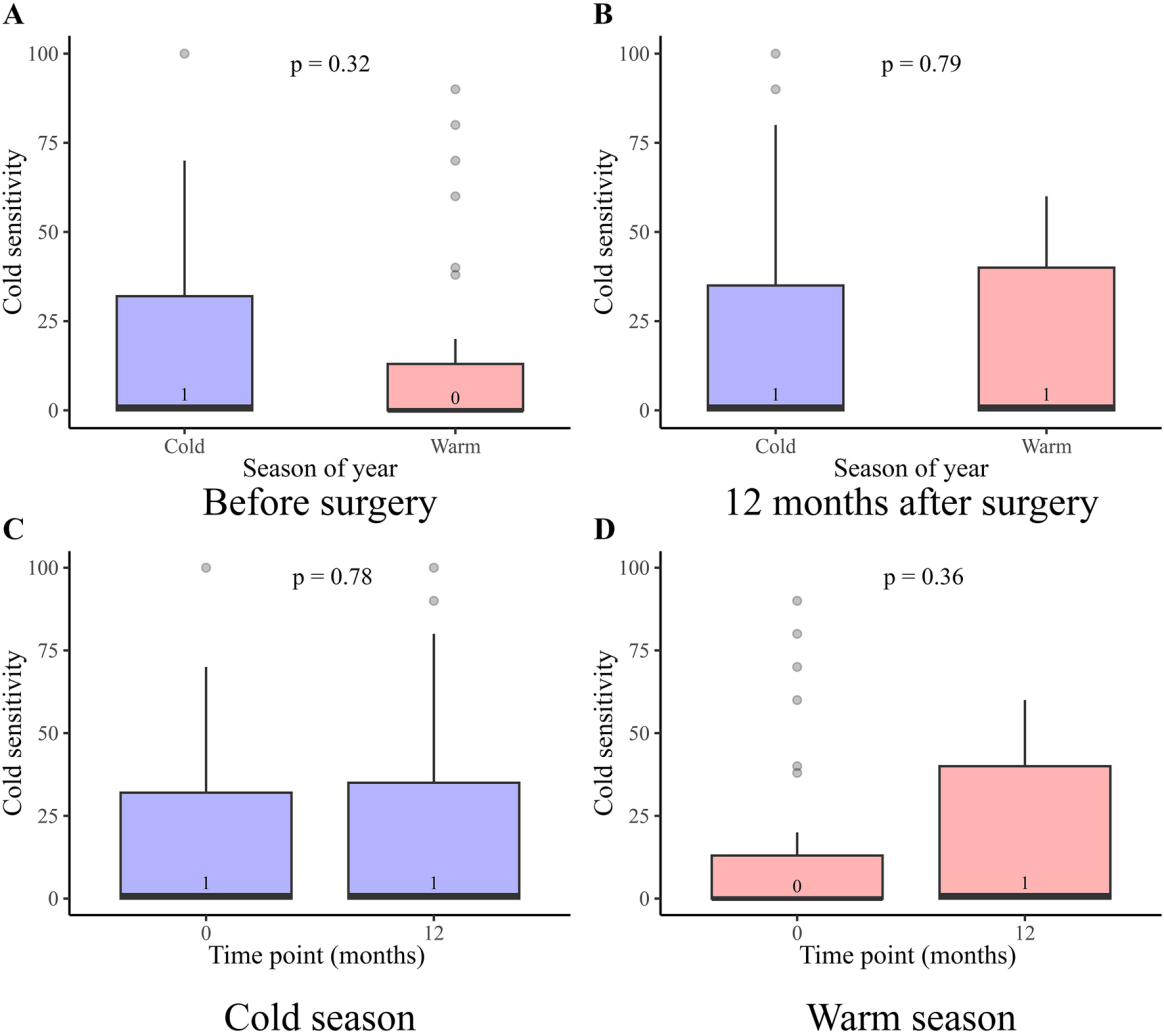
(**A**) Boxplot presenting cold sensitivity before surgery arranged by if surgical intervention was performed during the cold or warm season of the year in Sweden. (**B**) Boxplot presenting cold sensitivity 12 months after surgery arranged by if surgical intervention was performed during cold or warm season of the year in Sweden. (C) Boxplot presenting cold sensitivity before surgery and at 12 months after surgery if the surgical intervention was performed during the cold season (October–March) in Sweden. (D) Boxplot presenting cold sensitivity before and 12 months after surgery if surgical intervention was performed during the warm season (April-September) in Sweden. *p*-values in (**A**,**B**,**C**) and (**D**) are associated with Mann–Whitney U tests between groups in the respective figure.

QuickDASH total score was available at any time point in 111 out of 206 (54%) cases and the highest response rate was found before surgery (70/206, 34%) with a drop at three (45/206, 22%) and 12 months (61/206, 30%) postoperatively. The number of cases with complete data coverage before surgery and at 1 year follow-up were 26 out of 206 (13%). A significant decrease in QuickDASH score was found postoperatively (*p* < 0.001), where the post hoc analysis showed an improvement in QuickDASH score preoperatively to three months after surgery. However, no further change was found between three and 12 months after surgery (Fig. 1; Table 2). For HQ-8, the general response rate was 70/206 (34%) before surgery, 45/206 (22%) at three months, and 63/206 (31%) at 12 months postoperatively. Pain at rest, pain on motion without load, pain on load, and ability to perform daily activities all improved significantly over time (*p* < 0.001; Table 2; Fig. 2); again, with the significant change preoperatively to three months without any change between three and 12 months after surgery. Stiffness and cold sensitivity were unusual symptoms before and after surgery (Fig. 2D, G; Table 2). Further analysis of cold sensitivity, controlled for whether the surgical intervention was performed in the colder or warmer season of the year in Sweden, showed no significant differences in comparisons before and at 12 months after surgery (*p* = 0.32 and *p* = 0.79, respectively; Fig. 3A and B). In addition, no significant differences were found when comparing cold sensitivity before and at 12 months after surgery considering if the surgical intervention was performed during a cold or a warm season of the year (*p* = 0.78 and *p* = 0.36, respectively; Fig. 3C and D). Most of the participants declared no interference with social activities or described any difficulties to sleep after surgery. However, a statistically significant general improvement was found regarding both interference with social activities (*p* < 0.01) and difficulties to sleep (*p* < 0.001) (Table 2). Interference with social activities and difficulties to sleep improved three months after surgery (*p* < 0.05 for both), but no further improvements were noted between three and 12 months after surgery (*p* = 0.58 and *p* = 0.28, respectively).

**Table 2:** Descriptive statistics of examined questionnaire items and QuickDASH total score in patients surgically treated for peripheral nerve tumours in the upper limb.

Outcome	Time point		
	Before surgery	3 months*	12 months*
QuickDASH total score	18 [5–36], (n = 70)	5 [0–25], (n = 45)	5 [0–22], (n = 61)
Pain at rest	20 [0–40], (n = 70)	0 [0–16], (n = 45)	0 [0–10], (n = 63)
Pain on motion without load	20 [0–40], (n = 70)	0 [0–10], (n = 45)	0 [0–10], (n = 63)
Pain on load	24 [1–69], (n = 68)	10 [0–30], (n = 45)	1 [0–30], (n = 63)
Stiffness	0 [0–30], (n = 70)	0 [0–20], (n = 45)	0 [0–20], (n = 63)
Weakness	4 [0–40], (n = 70)	1 [0–31], (n = 45)	0 [0–30], (n = 62)
Numbness / tingling in fingers	11 [0–49], (n = 70)	10 [0–40], (n = 43)	10 [0–43], (n = 62)
Cold sensitivity	0 [0–30], (n = 70)	0 [0–20], (n = 37)	1 [0–40], (n = 63)
Ability to perform daily activities	11 [0–50], (n = 68)	0 [0–20], (n = 44)	0 [0–20], (n = 63)
Interference with social activities	1 [1–3], (n = 70)	1 [1, 2], (n = 45)	1 [1–1], (n = 63)
Severity of difficulties to sleep	1 [1–3], (n = 70)	1 [1–1], (n = 46)	1 [1–1], (n = 63)
Patient satisfaction (outcome after surgery)	**	9 [0–20], (n = 44)	0 [0–20], (n = 63)
Patient satisfaction (care at attending clinic)	**	0 [0–8], (n = 45)	0 [0–1], (n = 63)

Patient reported outcome measures with time point measurements presented as median, interquartile range, and number of responses. General response rate for QuickDASH preoperatively and at three, and 12 months were 70/206 (34%), 45/206 (22%) and 61/206 (30%), respectively. The corresponding values for HQ-8 were 70/206 (34%), 45/206 (22%), and 63/206 (31%). *Postoperatively; **Not applicable.

*Abbreviations* HQ-8, HAKIR questionnaire 8; QuickDASH, Quick Disabilities of the Arm, Shoulder and Hand.

### Patient perceived satisfaction

Patient satisfaction of perceived outcome after operation and the received care at the attending clinic was high at three and 12 months after surgery (Table 2).

### Correlations

**Figure 4:**
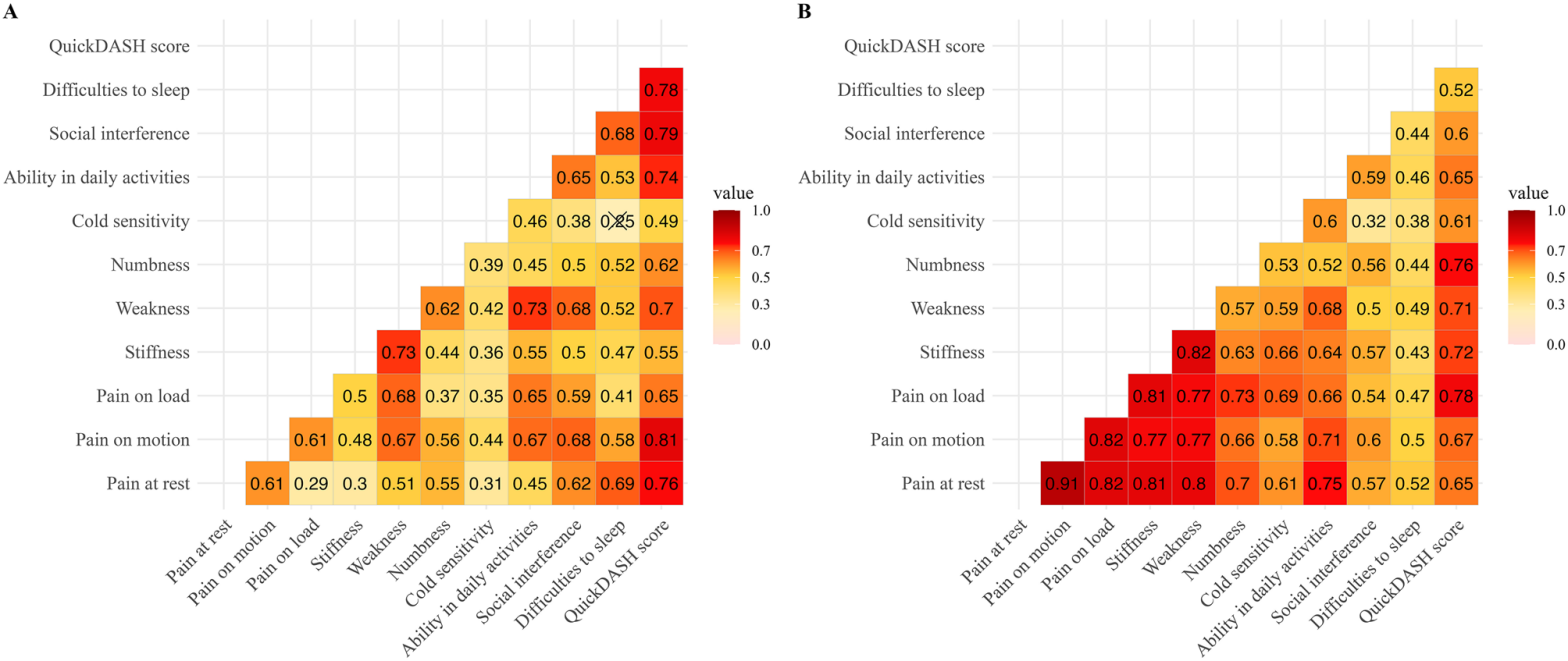
Spearman's correlation matrix with colour-graded and numerically displayed correlation coefficient (Spearman’s ρ) between PROMs collected before (**A**) and at 12 months after surgery (**B**). After False Discovery Rate (FDR)-correction (Benjamini–Hochberg Procedure), pre- and postoperative significance levels in correlations at different levels were found with *p* < 0.05. A statistically non-significant correlation is marked with a cross. *Abbreviations* QuickDASH (Quick Disabilities of the Arm, Shoulder and Hand).

Spearman’s correlation was performed between all items in the HQ-8 questionnaire as well as interference with social activities and severity of difficulties to sleep, together with the total QuickDASH-score before and at 12 months after surgery. All correlations were statistically significant after False Discovery Rate adjustment, except cold sensitivity to difficulties to sleep, before surgery (Fig. 4).

## Discussion

Surgical treatment of benign peripheral nerve tumours in the upper limb, excluding reoperations, generally improved symptoms and disability for affected patients with the largest benefit seen during the first 3three months after surgery and without further noticeable improvements at 12 months after surgery with high patient satisfaction. Surgery also improved specific symptoms related to the nerve tumour, such as pain at rest, pain on motion without load and pain on load as well as improved ability to perform daily activities. However, surgery did not generally introduce or affect cold sensitivity.

Reported pain modalities before surgery were the most substantial problem for patients with peripheral nerve tumours in the upper limb. Most participants with pain caused by peripheral nerve tumours in the upper limb is described to be improved or completely cured by surgical treatment^13,14,25,26^. Although several previous studies are limited by relatively small sample sizes, the reported effects of surgery on pain are in accordance with the current results that demonstrate a significant, and remaining, decrease in pain of all three modalities. Some patients reported no pain (i.e., score of 0) concerning the three pain modalities before surgery and, in accordance with the results of these pain modalities expressed as median [interquartile range], the number of patients that reported no pain increased after surgery. This was also applicable to the ability to perform daily activities, with an increasing number of patients reporting no impairment (score of 0) after surgery. The pain scores in the HQ-8 questionnaire, as well as the other items reflecting function of the affected hand, correlated strongly to the QuickDASH score, indicating that pain affects the participant´s individual ability to perform daily tasks, although the total QuickDASH score also reflects a generalised level of disability in the upper limbs. The minimal clinically important difference (MCID) in total QuickDASH score is estimated to be 7–16 points^27,28^ and the MCID regarding HQ-8 questionnaire items are not yet described. The present change in QuickDASH was 13 points, which falls within the reported range of MCID for QuickDASH, indicating a clinically meaningful improvement. This finding coheres with the HQ-8 scores clearly indicating an improvement with a clinical significance concerning the three pain modalities and ability to perform daily activities.

Preoperative cold sensitivity was uncommon and, more importantly, did not generally increase by the surgical intervention and was not affected by whether the surgical intervention was performed and followed up during the warm or cold season of the year. The scores of the preoperative cold sensitivity were low compared to other studies with subjects who underwent surgery for ulnar nerve entrapment or open carpal tunnel release^19–21^. The low present cold sensitivity score after surgery may be interpreted as a successful atraumatic surgical approach. However, approximately half of the present patients reported no cold sensitivity and a quartile reported scores as high as 30 and 40 (75th percentiles) before and at 12 months after surgery, respectively. As we conclude that cold sensitivity is generally a minor issue for these patients, the reported distribution is of clinical significance as a quartile of patients report moderate cold sensitivity, and with a few outliers reporting severe cold sensitivity^20^, which has not previously been highlighted. Sensory dysfunction, specified as numbness or tingling sensation in fingers, was experienced more before surgery and remained after surgery. Neurological symptoms after surgery of peripheral nerve tumours in the upper limb may be temporary^6,17,26,29^ and benefits regarding sensory function after surgical intervention have been reported^17,26,29^. The broader definition of sensory dysfunction in other studies compared to the present definition “Numbness/tingling in the fingers” may explain the discrepancy as well as the main locations of the nerve tumours in the present study^8,13,17,26,30^ with equal distribution of tumours in the median and ulnar nerves^2,13,14^. Furthermore, other studies indicate that peripheral nerve tumours in the upper limb less often impose motor dysfunction than sensory impairment^8,13,17^, a perspective that the present findings appear to support as stiffness and weakness were not significantly reported before surgery and did not appear to become prominent issues after surgery.

Patient satisfaction of perceived outcome after surgery and patient satisfaction of received care at the attending clinic were both high after surgical intervention (i.e., median score 0 at 12 months), which may be explained by the present results as well as by a low risk of worsening of symptoms^14,26^.

Limitations in this study were incomplete coverage of responses on PROM questionnaires. However, Stirling et al. could not find any significant differences in QuickDASH score between responders and predicted values of postoperative non-responders in patients undergoing hand surgery, despite a response rate of 55%^31^. Another limitation was the inability to further differentiate between various types of benign peripheral nerve tumours due to the appropriately used ICD-code. Furthermore, information regarding the nerve and the level of surgical engagement were not always derivable, depending on how the surgical codes (NCSP-S) were utilized and collected by the registry, why sub-analyses were not possible due to relative number of cases with uncertain levels as well as cases involving other nerves, and related to the subsequent reduction of statistical power. Strengths in this study were a multidimensional evaluation of symptoms before and after surgery with gathered data on a national level reducing the risk of selection bias^24^.

We conclude that surgical treatment of benign peripheral nerve tumours in the upper limb usually improve pain modalities and disability. Other symptoms were rather rare both before and after surgery, and patients were generally satisfied with outcome and received care.

## Data Availability

All data generated or analysed during this study are included in the current study. The datasets generated during and/or analysed during the current study are not publicly available due to ethical concerns, involving patient privacy as per the Swedish Ethical Review Authority.
